# BUILDING RESEARCH AND EVALUATION CAPACITY OF INTERGENERATIONAL INITIATIVES: EXAMPLES FROM THE FIELD

**DOI:** 10.1093/geroni/igae098.2017

**Published:** 2024-12-31

**Authors:** Cal Halvorsen

**Affiliations:** Washington University in St. Louis, St. Louis, Missouri, United States

## Abstract

Conducting evaluations to assess the impact of projects, services, and programs is often a daunting task for nonprofit and grassroots organizations. More so, developing research projects that document the need for the work of these social-purpose organizations can be difficult for staff and volunteers busy with day-to-day responsibilities. This presentation will showcase an ongoing academic-organizational partnership created to increase the research and evaluation capacity of a national nonprofit organization and its network of nascent and established grantees bringing older and younger people together to work on major social and environmental issues. Topics addressed will be establishing and cultivating trusting and transparent relationships, developing a scope of work, using an organization-centric research capacity-building approach, engaging students in these efforts, and more. Further, specific examples of products from these research and evaluation projects will be shown to illustrate how to navigate the conflicting needs of academics on the tenure-track with those of community-based organizations. The primary implications of this academic-organization partnership are twofold: First, this model, piloted with an organization focused on fostering new intergenerational initiatives, can be used by gerontological researchers in a variety of fields to benefit the community while aligning with established academic expectations; and second, leaders within these nascent intergenerational initiatives have now learned how to conduct basic program evaluations within their own organizations, which should ultimately lead to the strengthening of programs that bring the generations together.

